# Increased plasma endostatin and GDF15 in indolent non-Hodgkin lymphoma

**DOI:** 10.48101/ujms.v128.9392

**Published:** 2023-05-09

**Authors:** Josefin Hidman, Anders Larsson, Måns Thulin, Torbjörn Karlsson

**Affiliations:** a Department of Medical Science, Uppsala University, Uppsala, Sweden; b Centre for Clinical Research Västmanland, Västmanland County Hospital, Uppsala University, Uppsala, Sweden; c Department of Medicine, Västmanland County Hospital, Västerås, Sweden; d Department of Clinical Chemistry, Uppsala University Hospital, Uppsala, Sweden; e Department of Mathematics, Uppsala University, Uppsala, Sweden; f Department of Haematology, Uppsala University Hospital, Uppsala, Sweden

**Keywords:** Growth differentiation factor 15, endostatin, matrix metalloproteinase 9, neutrophil gelatinase-associated lipocalin, non-Hodgkin lymphoma, angiogenesis

## Abstract

**Background:**

Increased microvascular density correlates with more advanced disease and unfavorable overall survival in non-Hodgkin lymphoma (NHL), suggesting that angiogenesis is important for disease progression. However, studies of anti-angiogenic agents in NHL patients, have generally not shown favorable outcomes. The aim of this study was to investigate whether plasma levels of a subset of angiogenesis-associated proteins are increased in indolent B-cell derived NHL (B-NHL) and to investigate whether the levels differ between patients with asymptomatic versus symptomatic disease.

**Methods:**

Plasma levels of growth differentiation factor 15 (GDF15), endostatin, matrix metalloproteinase 9 (MMP9), neutrophil gelatinase-associated lipocalin (NGAL), long pentraxin 3 (PTX3), and galectin 3 (GAL-3) were measured by ELISA in 35 patients with symptomatic indolent B-NHL, 41 patients with asymptomatic disease, and 62 healthy controls. Bootstrap t-tests were used to assess the relative differences in biomarker levels between groups. Group differences were visualized using a principal component plot.

**Results:**

Mean plasma endostatin and GDF15 levels were significantly higher in symptomatic and asymptomatic lymphoma patients than in controls. Symptomatic patients had higher mean MMP9 and NGAL than controls.

**Conclusions:**

The finding of increased plasma endostatin and GDF15 in patients with asymptomatic indolent B-NHL suggests that increased angiogenic activity is an early event in indolent B-NHL disease progression.

## Introduction

Around 90% of all lymphomas are non-Hodgkin lymphoma (NHL), and 85–90% of these are derived from B-cells (B-NHL) (1). Non-Hodgkin lymphoma is subdivided into aggressive and indolent types. Indolent NHL comprises a heterogeneous group of lymphomas that are considered incurable, except for patients with localized disease who can be cured by radiotherapy. There is individual variation in the course of the disease; some lymphomas progress rapidly or transform to a more aggressive disease, but most patients with indolent NHL have a good prognosis in which the disease requires no treatment or intermittent active treatment only. Treatment of asymptomatic NHL patients does not prolong overall survival (OS) (2).

The most common types of indolent NHL are follicular lymphoma, small lymphocytic lymphoma, and marginal zone lymphoma (3). In follicular lymphoma symptomatic or high-burden disease is defined as any tumor mass >7 cm, three or more nodal sites >3 cm, splenomegaly below the umbilical line, pleural or peritoneal effusions, cytopenias, B-symptoms or >5000 circulating lymphoma cells/mm³; these findings and symptoms are indications for treatment. These criteria for symptomatic disease can be extrapolated to the management of patients with other types of indolent NHL (2, 4). For patients with more advanced disease meeting criteria for treatment, regimens are based on the backbone of an anti-CD20 agent, which in follicular lymphoma is combined with chemotherapy (2).

Angiogenesis is of importance for solid tumor progression and the process has been well characterized at the molecular level (5). It is also important in NHL, but there are conflicting data on the correlation between increased angiogenesis and prognosis (6). A recent meta-analysis suggests that increased angiogenesis, as evaluated by microvascular density (MVD), is associated with decreased OS in patients with indolent NHL (7).

In cancer, disturbances in the extracellular matrix (ECM) metabolism play an important role in the creation of a microenvironment favoring tumor growth and angiogenesis (8, 9). The ECM provides structural tissue support as well as exerting a variety of biochemical functions (9). Tumor cells promote degradation of the ECM, which stimulates angiogenesis via the release of cytokines such as vascular endothelial growth factor (VEGF) sequestered in the ECM and via matrix remodeling. Local tumor expansion is also dependent on proteolytic degradation of its surrounding ECM by matrix metalloproteinases (MMPs) (9). To grow beyond a certain volume, a malignant tumor is dependent on blood supply via newly formed vessels. The initiation of blood vessel expansion into the tumor is referred to as the angiogenic switch (10).

Therapies using anti-angiogenic agents in NHL patients have not shown the same favorable outcomes as seen in treatment of patients with solid tumors (6).

The aim of this study was to investigate plasma levels of six angiogenesis-associated proteins in an attempt to reveal their possible roles in indolent B-NHL progression. We have recently shown that the plasma level of growth differentiation factor (GDF15) is increased in multiple myeloma (MM) and in chronic lymphatic leukemia (CLL) (11, 12). Plasma matrix metalloproteinase 9 (MMP9) and the pro-angiogenic glycoprotein galectin 3 (GAL-3) are also increased in CLL (12). Increased levels of the angiogenesis-associated proteins endostatin, neutrophil gelatinase-associated lipocalin (NGAL), and long pentraxin 3 (PTX3) have been observed in different types of NHL, in CLL and in NK/T-cell lymphoma (13–15).

Plasma levels of GDF15, endostatin, MMP9, NGAL, PTX3, and GAL-3 were determined by ELISA and compared between patients with symptomatic indolent B-NHL, patients with asymptomatic disease, and healthy controls. This is to the best of our knowledge the first study investigating GDF15 in patients with indolent B-NHL.

## Material and methods

### Patients

Blood plasma drawn from 76 newly diagnosed indolent B-NHL patients and stored in the Uppsala-Umeå Cancer Consortium (U-CAN) biobank was used for ELISA analyses of GDF15, endostatin, MMP9, NGAL, PTX3, and GAL-3. Blood was sampled at the time of diagnosis. The patients were divided into two groups according to current guidelines (2, 4) – those with symptomatic lymphoma (*n* = 35) and those with asymptomatic disease (*n* = 41). Whether a patient was considered symptomatic or not was decided at the discretion of the treating physician. Laboratory and clinical data and pathology reports from the time of the lymphoma diagnosis were obtained from each patient’s individual chart. Of the 76 patients, 23 (30%) were diagnosed with follicular lymphoma, 23 (30%) with Waldenstrom’s macroglobulinemia, 6 (8%) with small lymphocytic lymphoma, 3 (4%) with marginal zone lymphoma, one with lymphoplasmacytic lymphoma and one with hairy cell leukemia, and 19 (25%) with indolent B-cell lymphoma not otherwise specified (NOS). Of the patients with B-cell lymphoma NOS one was diagnosed by splenectomy, five by lymph node biopsy or fine needle aspiration, eight by bone marrow aspiration or biopsy, one from peripheral blood, and four from biopsy of extra nodal tissue. Ten patients (13%) had stage I lymphoma according to the Ann Arbor staging system, 6 patients (8%) had stage II lymphoma, 10 (13%) had stage III lymphoma, and 50 (66%) had stage IV lymphoma. Blood plasma from 62 age- and sex-matched individuals was used as control. Written informed consent was obtained from all study participants. This study was approved by the Research Ethics Committee of Uppsala University and the Swedish Research Ethics Committee, respectively (Epn 2010/98, 2014/233, Ups-01367, and 2020-00164).

### Laboratory analyses

ELISA analyses were performed using commercial sandwich kits (R&D Systems, Minneapolis, MN, USA) for GDF15 (DY957), endostatin (DY1098), MMP9 (DY911), NGAL (DY1757), GAL-3 (DY1154), and PTX3 (DY1826). A monoclonal antibody specific for the different peptides was coated onto microtiter plates. Samples and standards were pipetted into the wells, after which the peptide was bound to the immobilized antibodies. A biotinylated peptide-specific antibody was added after washing. A streptavidin-HRP conjugate was added to the wells after incubation and washing and a substrate solution was added after another incubation and washing cycle. The absorbance was measured in a SpectraMax 250 (Molecular Devices, Sunnyvale, CA, USA). Concentrations were determined by comparing the optical density of the samples with the standard curve. All assays were calibrated against highly purified recombinant human peptides. Measurements were performed blinded, without knowledge of the clinical diagnoses.

### Statistics

Statistical analyses were calculated using version 4.2.1 of R (R Foundation for Statistical Computing, Vienna, Austria). To assess the relative differences in biomarker levels, age, C-reactive protein (CRP), albumin, creatinine, hemoglobin, and lactate dehydrogenase between groups, bootstrap t-tests were used. Group differences were visualized using a principal component plot (16). All *P*-values were adjusted for multiplicity using the Benjamini–Hochberg procedure (17).

## Results

Among the 76 lymphoma patients included in this study the majority had follicular lymphoma (30%) or Waldenstrom’s macroglobulinemia (30%), while 25% were diagnosed with low-grade B-NHL not otherwise specified. The median age for patients with symptomatic and asymptomatic lymphoma was 69 and 71 years, respectively. The control group consisted of 62 individuals with a median age of 60 years. The two groups of lymphoma patients did not differ significantly in terms of age, CRP, albumin, creatinine, hemoglobin, or lactate dehydrogenase (Table 1).

**Table 1:** Clinical and laboratory characteristics of patients with symptomatic and asymptomatic B-cell derived NHL. Age, hemoglobin, lactate dehydrogenase, albumin, creatinine, and C-reactive protein are expressed as median, range. N/A = not applicable.

Variable	Symptomatic B-NHL (*n* = 35)	Asymptomatic B-NHL (*n* = 41)	*P*
Age (years)	69 (40–89)	71 (41–92)	0.2
Male gender (%)	49	51	N/A
Hemoglobin (g/L)	124 (51–158)	127 (110–157)	0.31
Lactate dehydrogenase (µkat/L)	2.9 (1.8–7.1)	2.6 (1.5–6.9)	0.74
Albumin (g/L)	37 (16–49)	38 (30–45)	0.2
Creatinine (µmol/L)	66 (35–207)	64 (43–104)	0.31
CRP (mg/L)	2.1 (1.3–61)	1.8 (1.1–82)	0.64
Ann Arbor stage I (*n*)	8	2	N/A
Ann Arbor stage II (*n*)	4	2	N/A
Ann Arbor stage III (*n*)	4	6	N/A
Ann Arbor stage IV (*n*)	21	29	N/A
Follicular lymphoma (*n*)	16	7	N/A
Waldenstrom’s macroglobulinemia (*n*)	5	18	N/A
Small lymphocytic lymphoma (*n*)	0	6	N/A
Marginal zone lymphoma (*n*)	2	1	N/A
Lymphoplasmacytic lymphoma (*n*)	1	0	N/A
Hairy cell leukemia (*n*)	0	1	N/A
B-cell lymphoma not otherwise specified (*n*)	13	6	N/A

**Figure 1:**
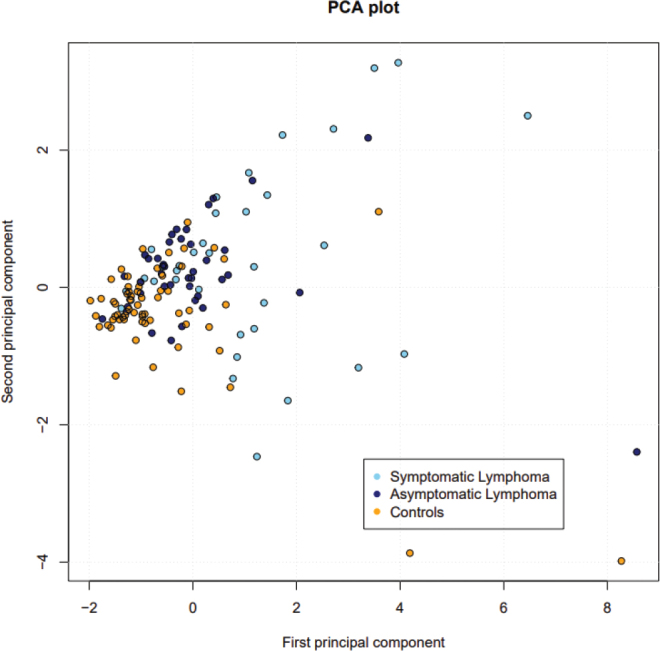
A PCA plot showing expression of the six plasma proteins studied. Controls (yellow) and patients with asymptomatic lymphoma (purple) have a more homogeneous expression pattern than patients with symptomatic lymphoma (blue), who express the proteins more heterogeneously.

A principal component analysis revealed that the expression patterns of the six plasma proteins studied were more homogenous in controls and in patients with asymptomatic lymphoma than in symptomatic patients, whose expression pattern was more heterogeneous (Figure 1).

Mean endostatin level was significantly higher in patients with symptomatic lymphoma than in controls (*P* < 0.01), and significantly higher in patients with asymptomatic disease than in controls (*P* < 0.01). There was no significant difference in mean endostatin between the two lymphoma patient groups (Figure 2a).

**Figure 2a:**
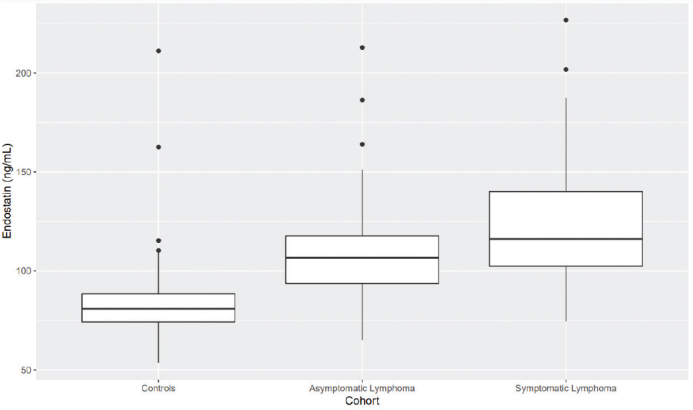
Plasma endostatin is significantly higher in patients with symptomatic and asymptomatic lymphoma than in controls (*P* < 0.01 in both cases).

Mean GDF15 level was significantly elevated in patients with symptomatic and asymptomatic lymphoma in comparison with controls (*P* < 0.05 in both cases), but did not differ significantly between the two patient groups (Figure 2b).

**Figure 2b:**
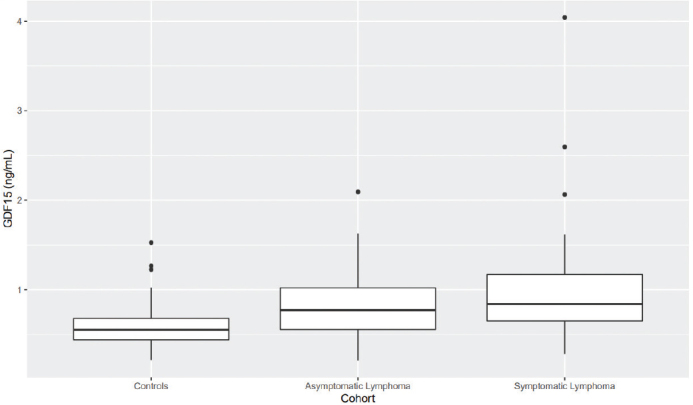
Plasma GDF15 is significantly higher in patients with symptomatic and asymptomatic lymphoma than in controls (*P* < 0.05 in both cases).

Patients with symptomatic lymphoma had significantly higher mean levels of MMP9 and NGAL than controls (*P* < 0.05 in both cases; Figures 2c–2d).

**Figure 2c:**
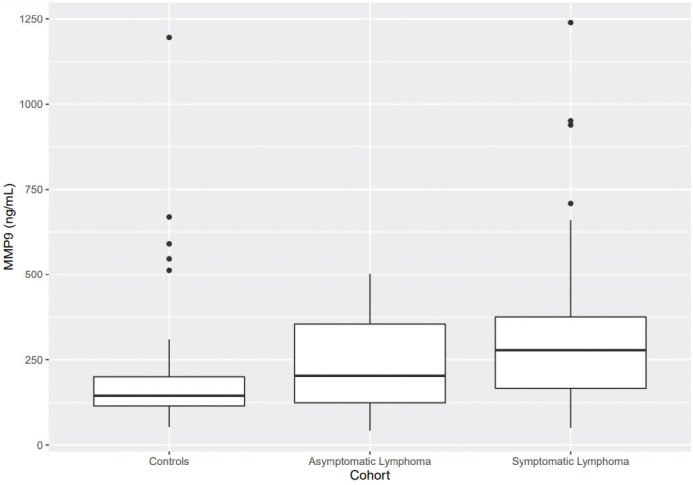
Patients with symptomatic lymphoma have significantly higher plasma MMP9 than controls have (*P* < 0.05).

**Figure 2d:**
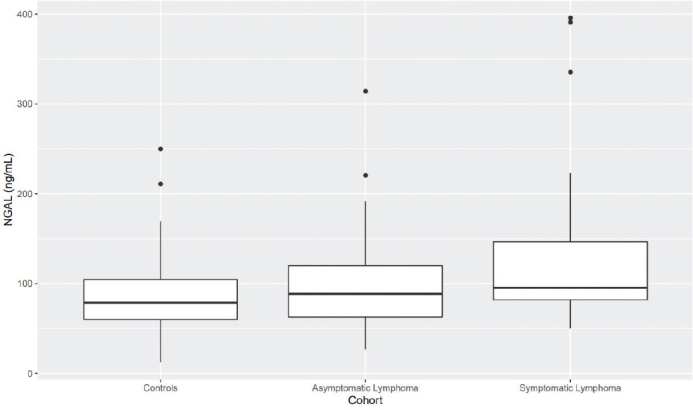
Patients with symptomatic lymphoma have significantly higher plasma NGAL than controls do (*P* < 0.05).

There were no significant differences in the mean levels of PTX3 and GAL-3 comparing the two lymphoma cohorts and controls (supplemental material, Figures 1–2)

## Discussion

Increased MVD in lymph nodes and bone marrow correlates with a more advanced disease stage and unfavorable OS in NHL, suggesting that angiogenesis is important for lymphoma progression (7). In an attempt to elucidate a possible role for increased angiogenesis in the transition of indolent B-NHL from an asymptomatic to a symptomatic state, we compared plasma levels of six angiogenesis-associated proteins between a group of patients with asymptomatic indolent B-NHL and a group of patients with symptomatic disease.

Figures 2a–d. Boxplots visualizing levels of GDF15, endostatin, MMP9, and NGAL in ng per mL, in controls and in patients with asymptomatic and symptomatic lymphoma. Medians are shown as thick lines, the bottoms and tops of the boxes represent the first and third quartiles, and the whiskers show the smallest and largest non-outliers. Outliers are shown as circles.

Principal component analysis revealed that patients with symptomatic B-NHL expressed these proteins more heterogeneously than both those with asymptomatic disease and controls (Figure 1). We have recently observed similar heterogeneous protein expression patterns of angiogenesis-related proteins in patients with CLL (12) and MM (Hidman, unpublished results) compared with controls. This similarity probably reflects the close biological relationship between indolent B-NHL, CLL, and MM.

Growth differentiation factor 15 is a pro-angiogenic protein that belongs to the transforming growth factor β superfamily. It is expressed in the placenta during physiological conditions as well as being secreted by activated macrophages in response to cellular stress signals such as inflammation, tissue injury, and hypoxia (18), and stimulates proliferation and migration of endothelial colony-forming cells (19). There are several other studies published showing that GDF15 stimulates angiogenesis *in vitro* (20, 21). Increased levels of circulating GDF15 are seen in patients with various types of cancer, for example, prostate and colorectal cancer (18). Growth differentiation factor 15 levels are also increased in MM and are associated with poor prognosis (11, 22, 23). Our finding of higher plasma GDF15 in patients with asymptomatic indolent B-NHL, compared with controls, points to an increased angiogenesis even in pre-symptomatic disease.

Matrix metalloproteinases are a family of proteolytic enzymes, which degrade components of the ECM. In cancer, MMPs are known to support tumorigenic processes such as angiogenesis, metastasis, proliferation, and invasion. Degradation of the ECM by MMPs promotes angiogenesis by several mechanisms including release of pro-angiogenic cytokines (such as VEGF) sequestered in the matrix. Matrix metalloproteinase activity is essential for angiogenesis and MMP inhibition reduces capillary growth (24). A study in rat skeletal muscle showed that decreased MMP activity was associated with a reduction in the number of microscopically observable capillary basement membrane breaks (25). Thus, it is conceivable that MMP activity facilitates vascular sprouting and capillary growth by making the vascular basement membrane permeable to migrating and proliferating endothelial cells. Matrix metalloproteinases are expressed in several types of solid tumors (24), and increased expression of MMP9 has also been seen in patients with CLL and high-grade NHL (12, 26).

Endostatin is formed by MMP proteolytic cleavage of type XVIII collagen. This type of collagen is abundantly expressed in basement membranes, including those in blood vessels (27). Increased circulating levels of endostatin are seen in patients with several different types of cancer (28). Endostatin exerts its effect on angiogenesis by binding VEGF receptors and inhibiting the angiogenic effect of VEGF, but the net effect on tumor angiogenesis is determined by the balance between anti- and pro-angiogenic factors (28). Since endostatin is formed by proteolytic degradation of collagen type XVIII by MMP9 (27), our observation of higher plasma endostatin in patients with asymptomatic lymphoma suggests that increased angiogenic activity is an early event in indolent B-NHL progression. Interestingly, we observed that endostatin was increased in plasma from asymptomatic B-NHL patients compared with controls, but MMP9 was not. There are several possible explanations for this. Firstly, we cannot exclude an increased MMP9 activity in asymptomatic patients, since we measured plasma MMP9 concentration and not protein enzymatic activity. Secondly, it is possible that the increased endostatin is a result of collagen XVIII degradation by proteases other than MMP9.

Neutrophil gelatinase-associated lipocalin, also known as lipocalin-2, is a protein expressed by neutrophils and multiple tissues in humans, including adipose, lymphatic, and respiratory tissue. Its expression is high in cancer tissue such as that from breast, pancreatic, and ovarian cancer (29). Moreover, treatment-naïve CLL patients have elevated levels of NGAL in serum (14). NGAL has the ability to form heterodimers with MMP9, thus preventing MMP9 degradation, and increased NGAL-MMP9 complex formation has been detected in certain cancers (29). Plasma NGAL and tumor NGAL mRNA have been shown to be increased in a murine hypoxic tumor model, and NGAL expression is increased in human and murine cancer cell lines cultured under hypoxic conditions (30). Neutrophil gelatinase-associated lipocalin stimulates angiogenesis *in vitro* by upregulation of VEGF expression, mediated by HIF-1α signaling (31). It has also been shown to promote angiogenesis in rodent brain endothelial cells, by iron and reactive oxygen species-related pathways (32). We found increased levels of plasma NGAL in patients with symptomatic, indolent B-NHL in comparison with controls, indicating that NGAL is involved in the increased angiogenesis observed in these patients.

Long pentraxin 3, an acute phase reactant, is involved in several aspects of cancer progression, such as angiogenesis and immune modulation (33). The pro-angiogenic glycoprotein GAL-3 is upregulated in anaplastic large cell lymphoma cells (34) and it has recently been shown that plasma GAL-3 is increased in CLL (12). In this study, we found no significant differences in mean plasma levels of PTX3 and GAL-3 when comparing the two lymphoma cohorts and the controls, indicating that these two proteins are not involved in indolent B-NHL angiogenesis and disease progression.

Despite previous findings of increased MVD correlating with a more advanced disease and decreased OS in patients with B-NHL, treatment with VEGF inhibitors and other anti-angiogenic agents has generally not shown the positive outcome seen in treatment of solid tumors (6). Lenalidomide is a potent inhibitor of angiogenesis *in vitro* (35), which could be an explanation for the positive effects on response rate and progression-free survival observed in relapsed indolent B-NHL when adding lenalidomide to rituximab (36). Further indications that angiogenesis is of importance for lymphoma progression include findings that addition of the VEGF-A antibody bevacizumab to rituximab treatment prolonged PFS in patients with relapsed follicular lymphoma (37), and that the anti-angiogenic multikinase inhibitor sorafenib given in monotherapy to patients with relapsed lymphoma has shown disease stabilization in patients with indolent B-NHL (38).

In summary, we found that four proteins related to angiogenesis, that is GDF15, endostatin, MMP9, and NGAL, are increased in plasma from patients with symptomatic B-NHL. This finding of increased GDF15 in patients with B-NHL has to our knowledge not previously been shown. Since we did not have access to bone marrow and lymph node biopsies from the lymphoma patients, we could not perform immunohistochemical analyses for vascular markers. This is an obvious limitation of our study. We suggest that increased plasma GDF15 reflects an increased angiogenic activity and not an inflammatory response primarily, since there was no difference in mean CRP between the two lymphoma patient cohorts (Table 1). It is possible that GDF15 could be a marker for more advanced disease since it is secreted in response to cellular stress, but the fact that we did not find a significant difference in levels of albumin, hemoglobin or lactate dehydrogenase between the two lymphoma patient groups makes this less probable (Table 1). The rationale behind our decision to compare protein expression between the two lymphoma cohorts was to identify angiogenesis-associated proteins that might be involved in the transition from asymptomatic to symptomatic disease. A comparison between the different lymphoma subtypes was not performed because of the limited number of patients. Blood samples were taken at diagnosis only, so no longitudinal follow up was done. These are also limitations of our study. Our discovery of increased plasma endostatin and GDF15 in asymptomatic indolent B-NHL indicates that increased angiogenic activity plays a role in the transition from asymptomatic to symptomatic lymphoma disease. It is possible that the increased endostatin reflects a transformation of the intratumoral capillary basement membranes to a more cell-permeable state, and that GDF15 stimulates endothelial cell migration through the breaks in capillary basement membranes and cell proliferation, creating new vessels. Hence, increased plasma endostatin and GDF15 probably reflect high intratumoral concentrations of these two molecules.

Previous studies on anti-angiogenic agents in patients with indolent B-NHL have only included patients with relapsed or refractory disease. The results of our study indicate increased angiogenic activity in an early disease stage, emphasizing that studies of anti-angiogenic treatment in patients with asymptomatic indolent B-NHL could be of interest.
