# Supplementary material for: Increased plasma endostatin and GDF15 in indolent non-Hodgkin lymphoma
Source: Ups J Med Sci. 2023 May 9;128:10.48101/ujms.v128.9392. doi: 10.48101/ujms.v128.9392 (PMC10202080; doi:10.48101/ujms.v128.9392)
Supplement: Supplementary file 1 [file UJMS-128-9392-s001.pdf]

Supplemental material

Figures 1–2. Boxplots visualizing levels of PTX3 and GAL-3 in ng per mL, in controls and in patients with asymptomatic and symptomatic lymphoma. Medians are shown as thick lines, the bottoms and tops of the boxes represent the first and third quartiles, and the whiskers show the smallest and largest non-outliers. Outliers are shown as circles.

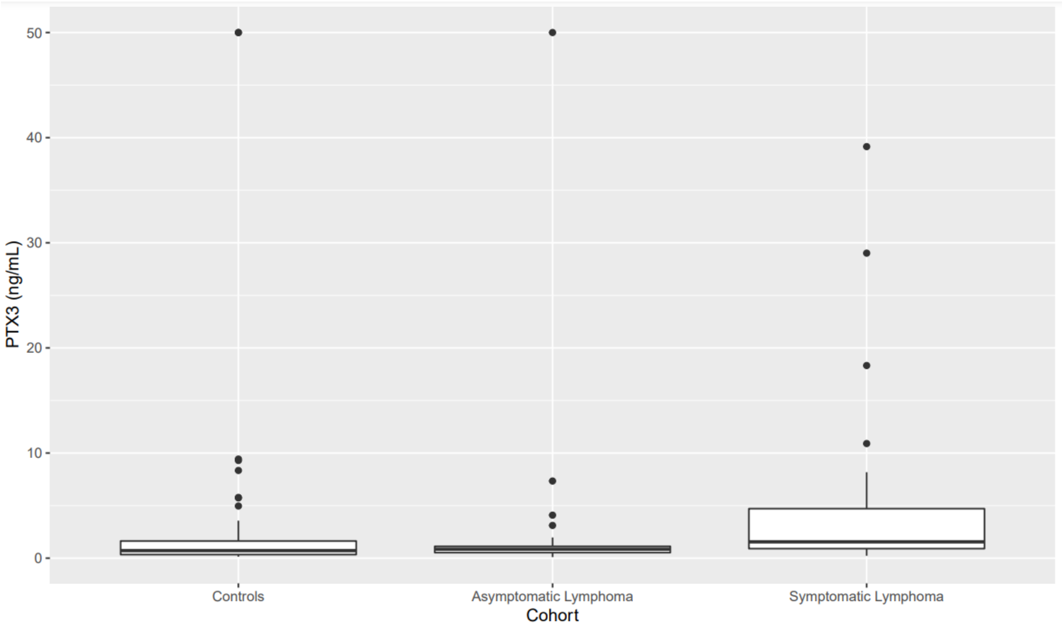

Figure 1. There is no significant difference in plasma levels of PTX3 comparing the two lymphoma cohorts and controls

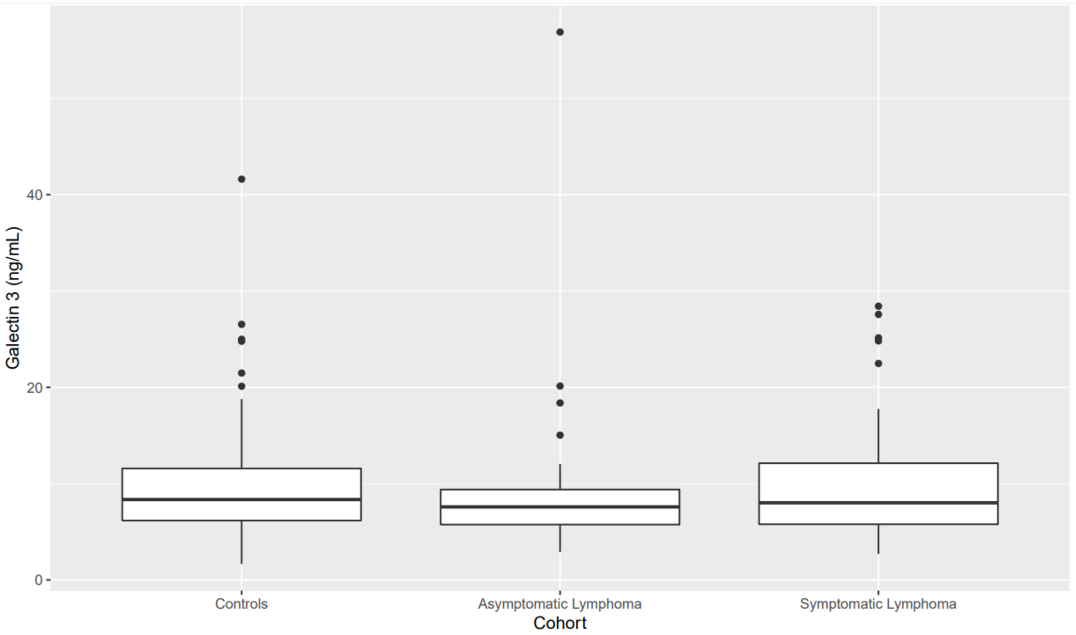

Figure 2. There is no significant difference in plasma levels of GAL-3 comparing the two lymphoma cohorts and controls
